# Real-world Outcome of Vosoritide Treatment in Children With Achondroplasia: A 12-month Retrospective Observational Study

**DOI:** 10.1210/jendso/bvaf041

**Published:** 2025-03-08

**Authors:** Susanna Reincke, Oliver Semler, Shino Junghänel-Welzing, Stefanie Stasek, Mirko Rehberg, Eva Pfeiffer, Heike Hoyer-Kuhn

**Affiliations:** Department of Pediatrics and Adolescent Medicine, Faculty of Medicine and University Hospital Cologne, University of Cologne, Cologne 50937, Germany; Department of Pediatrics and Adolescent Medicine, Faculty of Medicine and University Hospital Cologne, University of Cologne, Cologne 50937, Germany; Centre for Rare Skeletal Diseases in Childhood, Children's Hospital, University of Cologne, Cologne 50937, Germany; Department of Pediatrics and Adolescent Medicine, Faculty of Medicine and University Hospital Cologne, University of Cologne, Cologne 50937, Germany; Department of Pediatrics and Adolescent Medicine, Faculty of Medicine and University Hospital Cologne, University of Cologne, Cologne 50937, Germany; Department of Pediatrics and Adolescent Medicine, Faculty of Medicine and University Hospital Cologne, University of Cologne, Cologne 50937, Germany; Department of Pediatrics and Adolescent Medicine, Faculty of Medicine and University Hospital Cologne, University of Cologne, Cologne 50937, Germany; Department of Pediatrics and Adolescent Medicine, Faculty of Medicine and University Hospital Cologne, University of Cologne, Cologne 50937, Germany

**Keywords:** skeletal dysplasia, FGFR3, CNP-analog, growth, annualized growth velocity

## Abstract

**Context:**

Vosoritide is the first approved targeted therapy for achondroplasia (ACH) based on increased annualized growth velocity in clinical trials. The aim of our project was an assessment of the real-world setting and treatment with vosoritide.

**Design:**

This was a 12-month, retrospective observational study on an inception cohort of 34 patients with ACH treated with vosoritide.

**Patients and Methods:**

Thirty-four patients with ACH (22 males; aged 2.8 to 15.3 years at treatment initiation) who received vosoritide treatment for at least 12 months at a specialized clinic for skeletal dysplasia in childhood were included in the analysis. Auxological measurements at baseline and after 12 months of therapy were converted into disease-specific (ACH) and general population [Centers for Disease Control and Prevention (CDC)] z-scores. Physical function assessed by a 6-minute walk test was converted into z-scores and compared to an unaffected reference cohort.

**Results:**

After 12 months of treatment, both ACH and CDC height z-scores showed significant increases, with mean changes (mean ± SD) of 0.52 ± 0.35 and 0.38 ± 0.44, respectively (both *P* < .0001). The annualized growth velocity exceeded reference values for untreated children with ACH. No significant changes were observed in body mass index, upper to lower body segment ratio (sitting height/height), or head circumference. The 6-minute walking distance improved, with z-scores increasing from −2.00 ± 1.12 to −1.39 ± 1.23 (*P* = .0215).

**Conclusion:**

In a real-world setting, children with ACH showed significant improvements in growth and physical function after 12 months of treatment with vosoritide.

Achondroplasia (ACH) is an autosomal-dominant disorder caused by gain-of-function mutations in the fibroblast growth factor receptor 3 gene (*FGFR3*) [1, 2]. The gain-of-function activates the mitogen-activated protein kinase/extracellular signal-regulated kinase pathway, which ultimately causes decreased chondrocyte proliferation and differentiation [3]. This leads to impaired endochondral ossification, resulting in disproportioned short stature and characteristic syndromic stigmata such as macrocephaly and midface hypoplasia. Particularly during childhood and adolescence, there is a risk of clinically significant major complications, including recurrent middle ear infections, conductive hearing loss, foramen magnum stenosis, hydrocephalus internus, spinal stenosis, kyphosis, lordosis, and scoliosis [4]. With an estimated prevalence of 3.7 to 4.6 per 100 000 births, ACH is considered the most common skeletal dysplasia [5, 6]. Treatment of ACH requires multidisciplinary management including pediatric, orthopedic, and neurosurgical assessments and counseling [7-9]. The first targeting drug (vosoritide) treating the underlying pathophysiology of ACH was approved in 2021. Vosoritide, as a recombinant C-type natriuretic peptide analog, stimulates endochondral ossification by inhibiting the overactive *FGFR3* pathway in ACH [10]. In a phase 3 randomized, double-blind, placebo-controlled trial, children with ACH aged 5 years and older receiving vosoritide showed an increase in annualized growth velocity of 1.57 cm/year [11]. Based on data derived from initial studies that investigated selected patient cohorts, vosoritide was recently approved by the US Food and Drug Administration for children of all ages and by the European Medicines Agency for children over the age of 4 months. However, long-term results and real-world data regarding growth, safety, and ACH-related complications are still limited [12-15].

It is still unclear whether data from clinical trials can be directly applied to a patient cohort treated within the label of the drug but without the inclusion and exclusion criteria of a clinical trial. Therefore, we assessed retrospectively the effect of vosoritide in all children treated so far at our center. This observational study addresses the need for real-life data on the growth of children with ACH aged ≥2 to <16 years treated with vosoritide over a 12-month period and investigates our hypothesis that the annualized growth velocity would be similar to that observed in the phase 3 trial.

## Patients and Methods

This is a retrospective analysis of all patients with ACH who received vosoritide treatment for at least 12 months at the specialized clinic for skeletal dysplasia of University Hospital Cologne, Germany, between October 2021 and May 2024. Each patient received daily subcutaneous injections of vosoritide at a weight-dependent dose according to the product information [16]. Data were systematically collected during clinical follow-ups at the initiation of therapy; at week 4; and at months 3, 6, 9, and 12 and were prospectively entered into a monocentric disease-specific registry. By June 2024, the registry included data on 56 patients, of whom 34 (12 female, 22 male) had completed at least 12 months of treatment. None of these 34 patients participated in formal National Clinical Trial-registered clinical trials during the observational period. The cohort of 34 patients was categorized into 3 subgroups according to age at therapy initiation (≥2 to <5 years, ≥5 to <10 years, and ≥10 to <16 years) and 2 subgroups according to sex (male, female).

The primary objective was to assess differences in annualized growth velocity by comparing the cohort and its subgroups with age-, sex- and disease-specific reference values by Savarirayan et al [17] using z-scores. Annualized growth velocity was determined by calculating the ratio of height change (in cm) to the precise number of days between the baseline and 12-month measurements, normalized to a 365.25-day year. These values were subsequently converted to age-, sex-, and disease-specific z-scores by subtracting the reference mean and dividing by the SD provided in the reference dataset [17]. Secondary objectives were to investigate changes in z-scores for height, weight, sitting height/height ratio, and head circumference, as well as changes in 6-minute walking distance, radiological bone age, and quality of life.

Trained nurses conducted the auxological measurements and 6-minute walking tests during clinical visits, ensuring consistency in the methods used throughout the 12-month observation period. Height and sitting height were measured with a stadiometer, weight with a sitting scale, and head circumference standardized with a measuring tape. Z-scores for auxological data were calculated [18, 19] at baseline and at month 12 using the Box-Cox power transformation (L), the median (M), and the coefficient of variation (S) from Center for Disease Control and Preventions (CDC) growth charts [20] and disease-specific growth charts by Merker et al [21, 22]. The CDC growth charts were used in the vosoritide phase 3 trial and were chosen here to maintain consistency and ensure comparability of z-score changes. The ACH growth charts by Merker et al were utilized to assess potential height increases relative to untreated children with ACH. These charts were derived from a European cohort, with most data coming from Sweden and Germany, and thus provided a suitable reference group.

The 6-minute walking distance was measured on a standardized flat-floor course during clinical follow-ups at baseline and months 6 and 12. Z-scores for the 6-minute walking distance were calculated for baseline and month-12 data using reference means and SDs from an unaffected cohort [23].

Radiographic imaging of the left hand was performed at baseline and at month 12 to determine bone age using the Greulich and Pyle method. The change in bone age over 12 months was assessed by subtracting the individual bone age at baseline from the bone age at month 12, followed by calculating the mean change across participants. Additionally, the ratio of bone age to chronological age was calculated. In cases where an exact bone age could not be determined due to dissociated maturation of the carpal bones, the progress of bone age in the different carpal bones during the 12-month observational period was assessed.

Quality of life was assessed using the KIDSCREEN-52 questionnaire, completed by both patients and their caregivers. This questionnaire, designed for children aged 8 and older, consists of 52 items across 10 dimensions: Physical Well-Being, Psychological Well-Being, Moods and Emotions, Self-Perception, Autonomy, Parent Relations and Home Life, Financial Resources, Peers and Social Support, School Environment, and Bullying. The last 4 dimensions were primarily applicable to older schoolchildren, which led to limited data in these areas. Due to additional missing data in these dimensions that could not be retrieved given the retrospective nature of this study, an analysis of the last 4 dimensions was not appropriate. Therefore, only the first 6 dimensions were evaluated in this analysis. For patients aged 8 years and older and their caregivers, responses were analyzed using standardized scores (T-score) according to the KIDSCREEN-52 manual and compared to international reference data. For patients under 8 years of age and their caregivers, the questionnaire was analyzed using absolute scores, as recommended in the original publications [24-26].

### Statistics

Categorical data from the tables, z-scores, and standardized scores from the quality of life questionnaires were calculated using Excel version 16.87. All statistical analyses and graphical representations were conducted with GraphPad Prism version 10.3.0. For the statistical analysis of auxological data, the 6-minute walking test, and pretreatment vs posttreatment quality of life, only patients with both baseline and 12-month data were included. For the statistical analysis of the cohort's baseline quality of life in comparison to an international mean for unaffected children, all children with baseline data were included. The graphical presentation of quality of life over time included all patients, even those with missing baseline or 12-month data.

Normal distribution was tested using the Shapiro-Wilk-Test. For normally distributed data, mean changes were calculated with a 95% confidence interval, and significance was tested using paired *t*-test and 1-sample *t*-test, the latter applied to the analysis of annualized growth velocity and baseline quality of life data. For nonnormally distributed data, the Wilcoxon matched pairs signed-rank test was applied. A *P*-value of less than .05 was considered statistically significant. Normally distributed data in Tables 1 and 2 were described as mean values and SD. Nonnormally distributed values presented in Table 2 were expressed as medians and interquartile ranges.

**Table 1.** Clinical data at baseline

	≥2 to <5 years	≥5 to <10 years	≥10 to <16 years	Total cohort
**Participants, n (%)**	7 (20.6)	21 (61.8)	6 (17.6)	34 (100)
Female	3 (8.8)	8 (23.5)	1 (2.9)	12 (35.3)
** **Male	4 (11.8)	13 (38.2)	5 (14.7)	22 (64.7)
**Height, n (%)**	6 (18.8)	20 (62.5)	6 (18.8)	32 (100)
Mean [cm] (range)	80.7 (78.3-83.0)	99.9 (85.2-116.5)	112.4 (97.7-121.0)	
ACH z-scores, mean ± SD	0.36 ± 0.88	0.52 ± 1.44	−0.11 ± 1.33	0.37 ± 1.32
CDC z-scores, mean ± SD	−4.58 ± 0.62	−4.81 ± 1.09	−5.22 ± 1.04	−4.84 ± 1.00
**Weight, n (%)**	7 (21.2)	20 (60.6)	6 (18.2)	33 (100)
Mean [kg] (range)	13.5 (11.2-16.1)	20.3 (14.5-26.1)	29.0 (21.5-36.6)	
ACH z-scores, mean ± SD	0.39 ± 1.13	0.07 ± 0.98	−0.26 ± 0.94	0.08 ± 1.00
**BMI, n (%)**	6 (18.8)	20 (62.5)	6 (18.8)	32 (100)
Mean [kg/m^2^] (range)	21.2 (17.5-25.2)	20.4 (15.2-28.6)	22.8 (21.3-25.0)	21.0 (15.2-28.6)
ACH z-scores, mean ± SD	0.32 ± 1.88	−0.36 ± 1.45	−0.23 ± 0.67	−0.21 ± 1.41
**Sitting height/height, n (%)**	3 (15.0)	13 (65.0)	4 (20.0)	20 (100)
Mean (range)	0.69 (0.69-0.70)	0.65 (0.60-0.69)	0.66 (0.65-0.67)	0.66 (0.60-0.70)
ACH z-scores, mean ± SD	−0.14 ± 0.57	−1.73 ± 2.06	0.21 ± 0.65	−1.10 ± 1.89
**Head circumference, n (%)**	5 (25.0)	11 (55.0)	4 (20.0)	20 (100)
Mean [cm] (range)	54.4 (52.5-57.0)	56.3 (53.5-59.0)	56.8 (56.0-58.1)	
ACH z-scores, mean ± SD	−0.30 ± 0.71	−0.20 ± 0.84	−0.41 ± 0.31	−0.27 ± 0.71
**Ratio of bone age/chronological age, mean ± SD**	0.86 ± 0.25 (n = 2)	0.82 ± 0.16 (n = 8)	0.93 ± 0.18 (n = 4)	0.86 ± 0.17 (n = 14)
**Patients with ACH-related/growth-affecting surgeries before/during vosoritide treatment, n (male/female)**	1 (0/1)	13 (7/6)	3 (3/0)	17 (10/7)
Limb-lengthening surgery, n (male/female)	0	5 (2/3)	0	5 (2/3)
Corrective osteotomy, n (male/female)	0	2 (1/1)	1 (1/0)	3 (2/1)
Foramen magnum decompression, n (male/female)	1 (0/1)	10 (5/5)	1 (1/0)	12 (6/6)
Ventriculoperitoneal shunt surgery, n (male/female)	0	1 (1/0)	1 (1/0)	2 (2/0)
Corrective spinal fusion surgery, n (male/female)	0	0	1 (1/0)	1 (1/0)
**General medical conditions, n (male/female)**	2 (2/0)	11 (6/5)	0	13 (8/5)
Intermittent low vitamin D levels requiring supplementation, n (male/female)	1 (1/0)	7 (4/3)	0	8 (5/3)
Nephrocalcinosis, n (male/female)	1 (1/0)	0	0	1 (1/0)
Hereditary spherocytosis, n (male/female)	0	1 (1/0)	0	1 (1/0)
Hearing impairment requiring hearing aids, n (male/female)	0	1 (0/1)	0	1 (0/1)
Prematurity, n (male/female)	0	1 (0/1)	0	1 (0/1)

Abbreviations: ACH, achondroplasia; BMI, body mass index; CDC, Centers for Disease Control and Prevention.

**Table 2.** Auxological data at baseline and month 12, mean and median changes after 12 months of therapy, corresponding significance values, and number of patients per subgroup

	Month 0	Month 12	Δ0-12 ± SD of differences	*P*-value	n
**Height CDC z-scores, mean ± SD**	−4.84 ± 1.00	−4.46 ± 1.06	0.38 ± 0.44	<.0001	32
Female	−4.47 ± 0.87	−4.13 ± 0.84	0.34 ± 0.42	.0218	11
Male	−5.04 ± 1.03	−4.63 ± 1.14	0.41 ± 0.46	.0006	21
≥2 to <5	−4.58 ± 0.62	−4.20 ± 1.00	0.37 ± 0.50	.1287	6
≥5 to <10	−4.81 ± 1.09	−4.43 ± 1.11	0.37 ± 0.44	.0012	20
≥10 to <16	−5.22 ± 1.04	−4.79 ± 1.03	0.43 ± 0.44	.0619	6
Patients with limb-lengthening surgery	−3.37 ± 0.93	−3.12 ± 0.81	0.26 ± 0.21	.0884	4
**Height ACH z-scores, mean ± SD**	0.37 ± 1.32	0.89 ± 1.37	0.52 ± 0.35	<.0001	32
Female	0.92 ± 1.00	1.38 ± 0.95	0.47 ± 0.29	.0003	11
Male	0.09 ± 1.40	0.64 ± 1.50	0.55 ± 0.38	<.0001	21
≥2 to <5, median (IQR)*^a^*	0.12 (−0.28; 0.84)	0.51 (0.26; 1.55)	0.68 (0.05; 1.03)	.0625	6
≥5 to <10	0.52 ± 0.99	0.99 ± 1.47	0.47 ± 0.28	<.0001	20
≥10 to <16	−0.11 ± 1.33	0.49 ± 1.31	0.60 ± 0.37	.0100	6
Patients with limb-lengthening surgery	2.38 ± 1.10	2.80 ± 1.03	0.42 ± 0.25	.0443	4
**Weight ACH z-scores, mean ± SD**	0.08 ± 1.00	0.30 ± 1.04	0.22 ± 0.36	.0013	33
Female	0.84 ± 0.77	1.01 ± 0.66	0.17 ± 0.47	.2547	11
Male, median (IQR)*^a^*	−0.43 (−1.03; 0.37)	−0.31 (−0.93; 0.52)	0.18 (0.06; 0.44)	.0012	22
≥2 to <5	0.39 ± 1.13	0.71 ± 1.25	0.32 ± 0.41	.0864	7
≥5 to <10	0.07 ± 0.98	0.28 ± 1.01	0.21 ± 0.37	.0231	20
≥10 to <16	−0.26 ± 0.94	−0.10 ± 0.84	0.16 ± 0.30	.2382	6
**BMI, mean [kg/m^2^] (range)**	21.0 (15.2-28.6)	21.2 (15.4-30.5)	NA	NA	32
**BMI ACH z-scores, mean ± SD**	−0.21 ± 1.41	−0.32 ± 1.36	−0.11 ± 0.46	.1802	32
Female, median (IQR)*^a^*	0.82 (−0.64; 1.25)	0.533 (−0.73; 0.85)	−0.09 (−0.59; 0.36)	.4131	11
Male	−0.46 ± 1.32	−0.58 ± 1.38	−0.11 ± 0.39	.1954	21
≥2 to <5	0.32 ± 1.88	0.08 ± 1.88	−0.24 ± 0.61	.3837	6
≥5 to <10	−0.36 ± 1.45	−0.42 ± 1.39	−0.05 ± 0.46	.6074	20
≥10 to <16	−0.23 ± 0.67	−0.41 ± 0.61	−0.18 ± 0.32	.2326	6
**Sitting height/height mean, range**	0.66 (0.60-0.70)	0.66 (0.61-0.70)	NA	NA	20
**Sitting height/height ACH z-scores, median (IQR)** *^a^*	−0.38 (−2.60; 0.13)	−0.41 (−1.58; 0.69)	0.30 (−0.18; 0.89)	.1327	20
Female, median (IQR)*^a^*	−0.49 (−2.95; −0.24)	−0.96 (−1.59; 0.76)	0.52 (−0.59; 1.25)	.3750	7
Male, median (IQR)*^a^*	−0.08 (−2.58; 0.36)	−0.12 (−1.87; 0.68)	0.22 (−0.15; 0.71)	.2163	13
≥2 to <5, mean ± SD	−0.14 ± 0.57	−0.38 ± 1.21	−0.24 ± 1.15	.7497	3
≥5 to <10, mean ± SD	−1.73 ± 2.06	−1.28 ± 1.99	0.45 ± 0.64	.0256	13
≥10 to <16, mean ± SD	0.21 ± 0.65	0.31 ± 0.47	0.10 ± 0.25	.4817	4
**Head circumference ACH z-scores, mean ± SD**	−0.27 ± 0.71	−0.15 ± 0.80	0.12 ± 0.39	.1763	20
Female	−0.36 ± 0.50	−0.23 ± 0.89	0.13 ± 0.59	.5418	8
Male	−0.21 ± 0.84	−0.09 ± 0.78	0.12 ± 0.20	.0719	12
≥2 to <5	−0.30 ± 0.71	−0.21 ± 0.85	0.93 ± 0.33	.5669	5
≥5 to <10	−0.20 ± 0.84	0.96 ± 0.24	0.09 ± 0.48	.5656	11
≥10 to <16	−0.41 ± 0.31	−0.15 ± 0.24	0.26 ± 0.10	.0124	4

Abbreviations: ACH, achondroplasia; BMI, body mass index; CDC, Centers for Disease Control and Prevention; IQR, interquartile range; NA, not applicable.

^a^All subgroups were tested for normal distribution using the Shapiro-Wilk test. In this table, normally distributed data is displayed as mean ± SD; nonnormally distributed data is displayed as median (IQR). For normally distributed data, significance was tested using paired *t*-test; for nonnormally distributed data, the Wilcoxon matched-pairs signed-rank test was applied.

## Results

### Clinical Data

Thirty-four patients with ACH (12 females and 22 males) were included in the analyses. Clinical data for these patients are presented in Table 1. All patients showed the same disease-causing mutation in *FGFR3* [c.1138G > A (p.Gly380Arg)]. The mean age at start of therapy was 7.52 years, with the youngest patient being 2.83 years old and the oldest 15.25 years old. The mean bone age/chronological age ratio at baseline was 0.86 ± 0.17 (n = 14), indicating a mean delayed bone age. The mean height z-score at the initiation of therapy was 0.37 ± 1.32 (mean ± SD) for ACH-specific percentiles, respectively −4.84 ± 1.00 for CDC percentiles.

We identified 17 patients who had undergone ACH-related and growth-affecting surgeries before or during the observational period. Of these, 5 patients had previously undergone limb-lengthening surgery prior to starting vosoritide, and 1 of these patients had additional limb lengthening during the observation period. Thirteen patients also presented with other medical conditions, as listed in Table 1. None of the 34 patients discontinued or interrupted the therapy within the 12-month period. However, vosoritide treatment of 1 patient was discontinued after 15 months, as decided by the family in agreement with the medical team, due to a lack of therapeutic benefit (5.7 years at therapy start, change in height ACH z-score of −0.09 after 12 months, individual annualized growth velocity of 3.17 cm/year, corresponding to a z-score of −0.73) and the patient's individual burden.

### Annualized Growth Velocity

Annualized growth velocity was assessed for 31 out of 34 patients. Two patients were excluded from this analysis due to incomplete data. One patient lacked baseline height measurements, and the other 1 had missing 12-month height data due to a recent limb-lengthening surgery, which prevented the standardized standing height measurement. Additionally, for 1 patient, no age-specific reference values were available, as these were only applicable up to 14 years of age and the patient was already 16 years old. Consequently, these 3 patients were excluded from the analyses of annualized growth velocity (but were included in the analysis of other auxological measurements).

Children aged ≥10 to <16 years (n = 5) exhibited the highest mean absolute growth velocity at 6.98 ± 1.44 cm/year, while those aged ≥5 to <10 years (n = 20) showed the lowest at 5.77 ± 1.24 cm/year. Children aged ≥2 to <5 years (n = 6) showed a mean growth velocity of 6.27 ± 1.68 cm/year. According to sex, females (n = 11) exhibited a higher mean absolute growth velocity (6.24 ± 1.04 cm/year) compared to males (n = 20) (5.97 ± 1.56 cm/year). Patients who had undergone previous limb-lengthening surgeries showed an annualized growth velocity of 5.55 ± 0.72 cm/year (n = 4).

**Figure 1.**
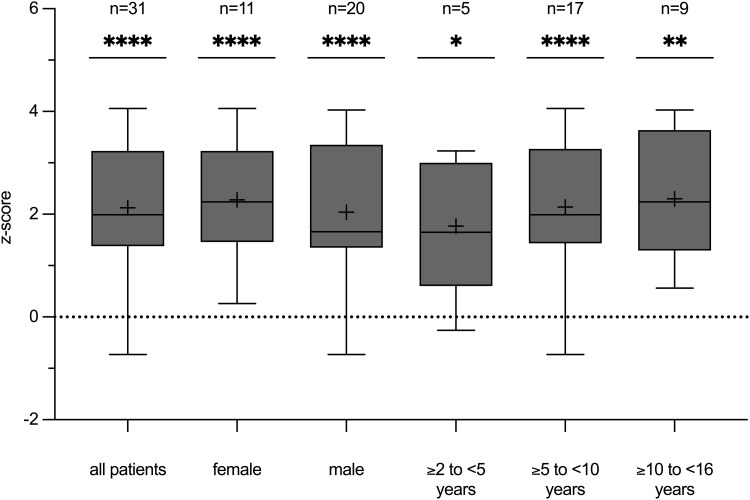
Box and whisker plots of posttreatment growth velocity z-scores. Z-scores were calculated using growth velocity reference values of an untreated achondroplasia cohort [17]. Each box plot displays the 25th and 75th quartiles (box edges), the median (midline), the mean (plus symbol), and the Tukey whiskers of the total cohort or of sex-/age-related subgroups. The individual annualized growth velocity was calculated as [(height_2_ − height_1_)/(date of measurement_2_ − date of measurement_1_)]/365.25 and converted into age-, sex-, and disease-specific z-scores by calculating [(individual annualized growth velocity − annualized growth velocity_reference_)/SD_reference_]. Individuals with an annualized growth velocity z-score lower than the reference mean are displayed below zero (dotted line). *P ≤ .05, **P ≤ .01, ***P ≤ .001, ****P ≤ .0001. *P* > .05 = not significant.

Notably, the individual annualized growth velocities were significantly higher compared to age-, sex-, and disease-specific reference values (Fig. 1). The cohort's mean z-score was 2.13 ± 1.26 (*P <* .0001). (The highest z-score was observed in the oldest age group (≥10 to <16 years) at 2.30 ± 1.23 (*P* = .0005), followed by the ≥5- to <10-year age group at 2.14 ± 1.32 (*P <* .0001) and the ≥2- to <5-year age group at 1.77 ± 1.36 (*P* = .0434). Annualized growth velocity according to sex revealed a significant z-score increase of 2.28 ± 1.14 (*P* = .0001) in females and of 2.04 ± 1.35 (*P* = .0001) in males. Additionally, patients who had undergone previous limb-lengthening surgeries demonstrated a z-score increase of 2.67 ± 1.38 (*P* = .0304, n = 4).

### Changes in Height z-scores

**Figure 2.**
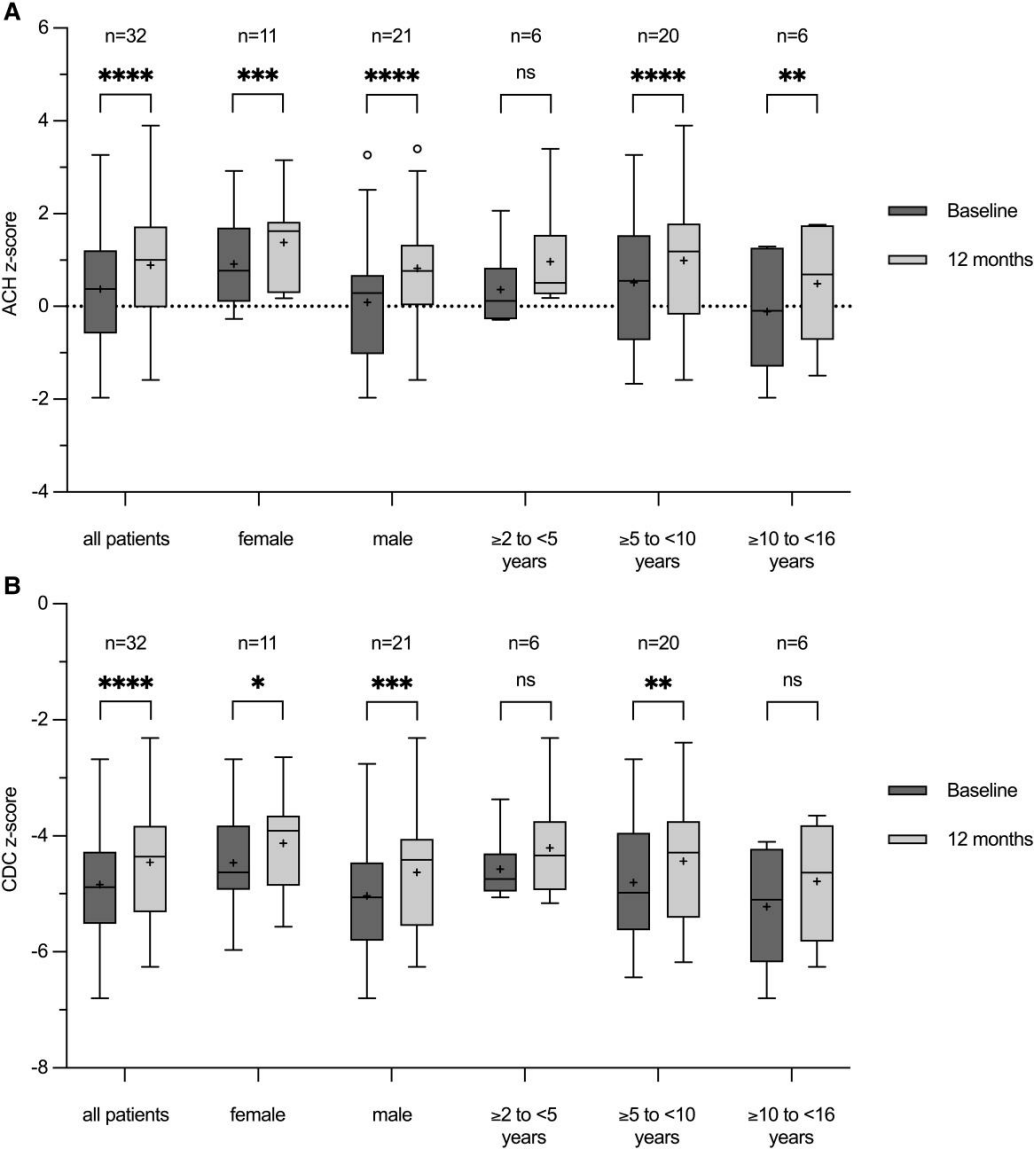
Box and whisker plots of height z-scores at baseline (darker grey, left) and at month 12 (lighter grey, right). Each box plot displays the 25th and 75th quartiles (box edges), the median (midline), the mean (plus symbol), and the Tukey whiskers of the total cohort or of sex-/age-related subgroups. Outliers are represented as circles. Z-scores were calculated using LMS data derived from auxological data of a European cohort with achondroplasia [21, 22] (A) and of an unaffected cohort, the latter provided by the Centers for Disease Control and Prevention [20] (B). *P ≤ .05, **P ≤ .01, ***P ≤ .001, ****P ≤ .0001. *P* > .05 = not significant.

Height measurements from 32 patients were converted into ACH-specific z-scores [21] and CDC z-scores [20] (Fig. 2A and 2B, Table 2). After 12 months of therapy, 29 of the 32 patients demonstrated improvement in their ACH z-score and 26 of the 32 patients showed an increase in CDC z-score. The mean ACH z-score for height after 12 months of therapy was 0.89 ± 1.37, which was significantly higher (*P <* .0001) than the mean z-score at the start of therapy (0.37 ± 1.32), resulting in a mean change in ACH height z-score of 0.52 ± 0.35. Similarly, the CDC z-scores showed a significant increase (*P <* .0001) from −4.84 ± 1.00 to −4.46 ± 1.06 over the 12-month therapy period, giving a mean change of 0.38 ± 0.44. Patients who had undergone limb-lengthening surgeries showed a mean change of 0.42 ± 0.25 (*P* = .0443, n = 4) in ACH z-score and 0.26 ± 0.21 (*P* = .0884, n = 4) in CDC z-score, reflecting a smaller improvement compared to the rest of the cohort.

Despite the positive mean change for the entire cohort, 3 out of 32 patients exhibited a slight decrease in their ACH z-score, with a mean decrease of −0.06 ± 0.03 after 12 months of therapy.

### Weight, Body Mass Index, Sitting Height/Height Ratio, and Head Circumference

**Figure 3.**
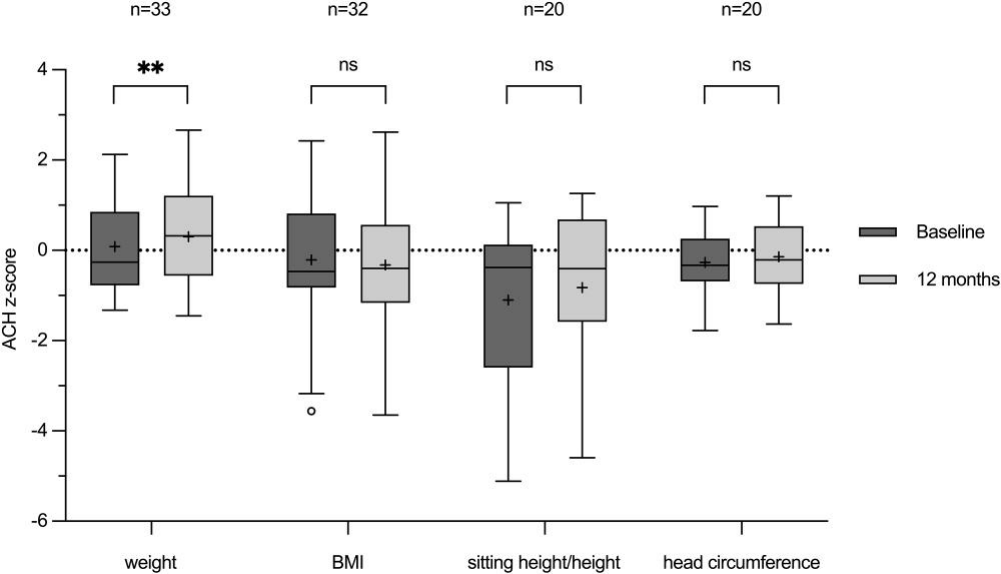
Box and whisker plots of achondroplasia z-scores of body weight, body mass index, sitting height/height and head circumference at baseline (darker grey, left), and at month 12 (lighter grey, right). Each box plot displays the 25th and 75th quartiles (box edges), the median (midline), the mean (plus symbol), and the Tukey whiskers of the total cohort or of sex-/age-related subgroups. z-scores were calculated using LMS data derived from auxological data of a European cohort with achondroplasia [21, 22]. *P ≤ .05, **P ≤ .01, ***P ≤ .001, ****P ≤ .0001. *P* > .05 = not significant.

Alongside the increased growth, there was a significant increase (*P* = .0013) in the ACH z-score for weight, from 0.08 ± 1.00 to 0.30 ± 1.04. However, no significant changes in body mass index or in head circumference or body proportions (sitting height/height) were observed after 12 months of therapy (Fig. 3, Table 2).

### 6-minute Walking Distance

**Figure 4.**
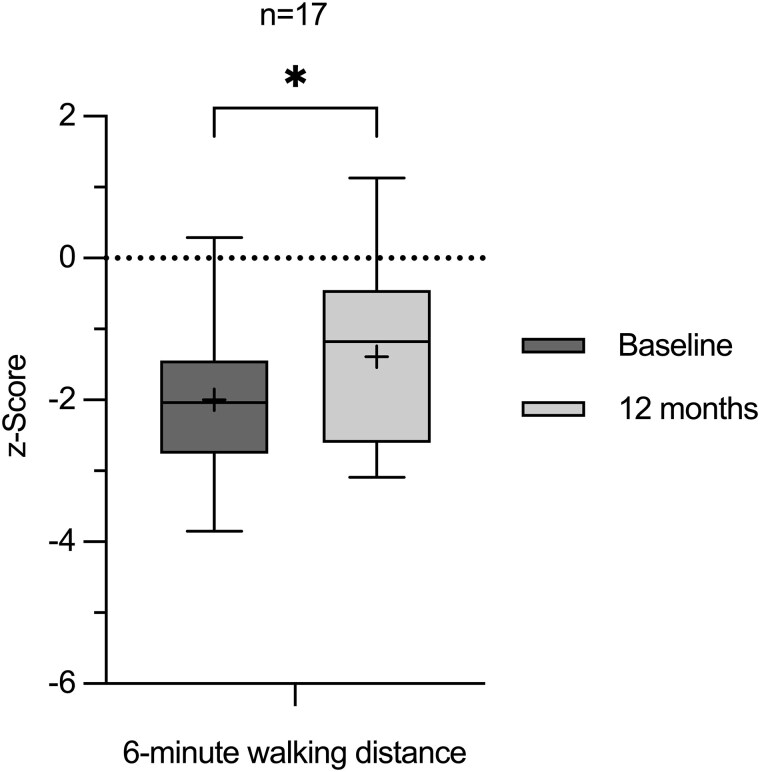
Box and whisker plots of z-scores of 6-minute walking test at baseline (darker grey, left) and at month 12 (lighter grey, right). Each box plot displays the 25th and 75th quartiles (box edges), the median (midline), the mean (plus symbol), and the Tukey whiskers of the total cohort or of sex-/age-related subgroups. z-scores were calculated using LMS data derived from auxological data of an unaffected cohort [23]. *P ≤ .05, **P ≤ .01, ***P ≤ .001, ****P ≤ .0001. *P* > .05 = not significant.

The 6-minute walking distances were converted into z-scores based on age-specific reference values from an unaffected cohort [23]. Aligning with the increased growth in height, a simultaneous notable increase (*P* = .0215) in z-scores for the 6-minute walking distance was observed, improving from −2.00 ± 1.12 to −1.39 ± 1.23 (Fig. 4).

### Bone Age

Bone age could be determined for 14 patients at baseline and 12 months. Over the 12-month period, the mean change in bone age was 1.01 ± 0.63 years (mean ± SD, n = 14). Consistent with this, the bone age to chronological age ratio remained unchanged, with a ratio of 0.86 ± 0.17 at baseline and 0.86 ± 0.15 (n = 14) at month 12. For the other patients, an exact bone age could not be determined due to dissociated maturation of the carpal bones. However, manual comparison of the radiographs revealed no signs of accelerated bone maturation during the observational period.

### Quality of Life

**Figure 5.**
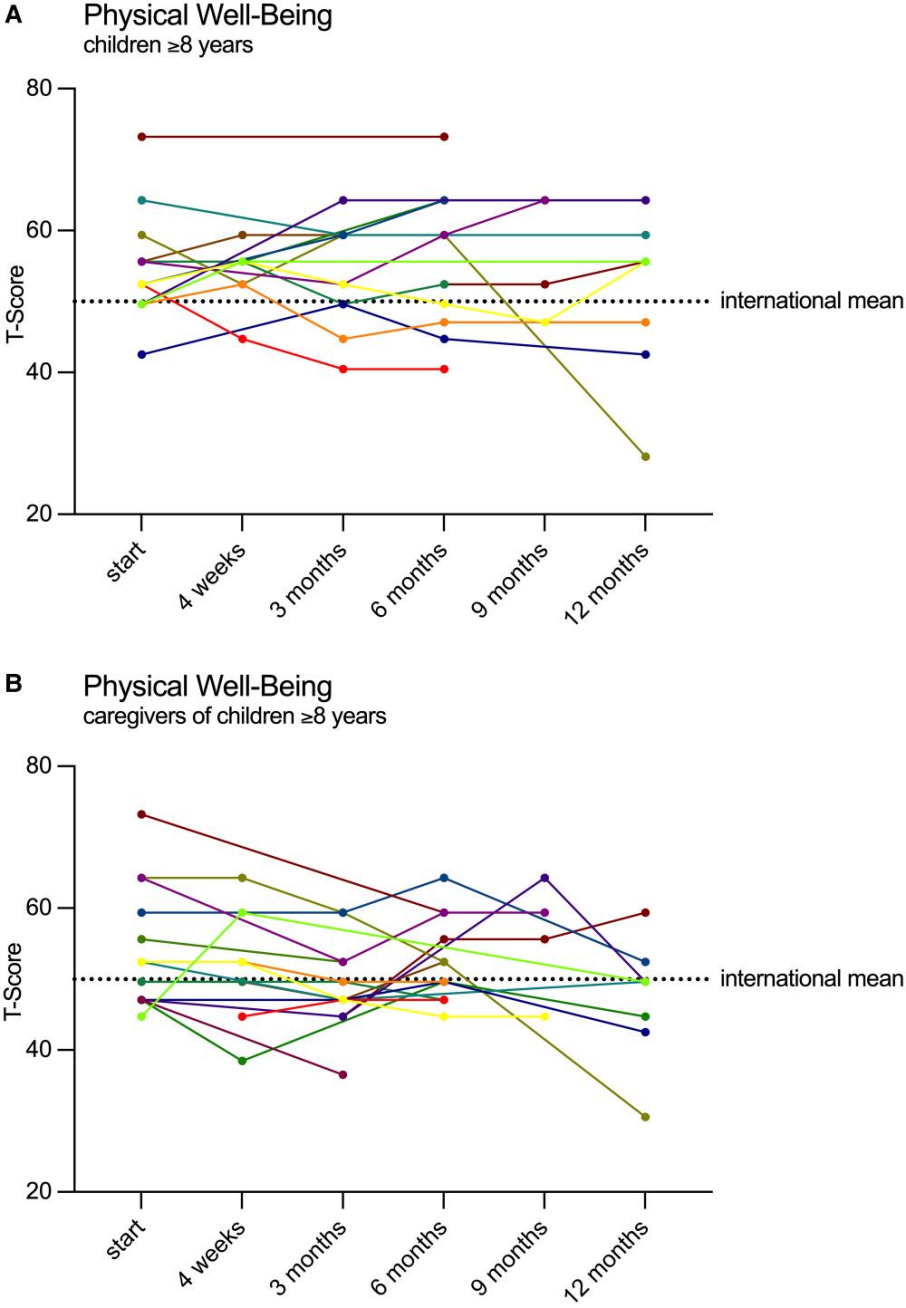
Time course of quality of life in the physical well-being dimension, determined by the KIDSCREEN-52 questionnaire. Responses were given by patients ≥8 years (A) and their caregivers (B). Each graph represents an individual patient and each dot the response given at that time. Responses were normalized and represented as T-scores according to the KIDSCREEN-52 manual. The dotted line symbols the international mean of an unaffected cohort in this dimension [24-26].

Quality of life was assessed using the KIDSCREEN-52 questionnaire, completed by both patients and their caregivers. All available data were graphically represented and analyzed over time. For children aged 8 years and older (and their caregivers), international reference values from an unaffected cohort were available. Interestingly, at the beginning of therapy, children with ACH aged 8 years and older scored significantly above the international mean in the Physical Well-Being dimension (Fig. 5A). Additionally, in the Self-Perception dimension, caregiver responses indicated scores significantly below the international mean, whereas the children's self-reported scores were above the mean, though not significantly (data not shown). Over time, no clear or significant trends were observed in the responses, although there was a slight downward trend in Physical Well-Being, especially in the responses by caregivers (Fig. 5B). Overall, the trajectories varied widely both between and within individuals.

### Reported Side Effects

Throughout the 12-month treatment period, 12 of the 34 patients reported mild local injection site reactions as adverse effects. Two patients experienced dizziness as a moderate side effect.

## Discussion

This study evaluated the effects of vosoritide treatment on the growth of children with ACH in a real-world setting. Consistent with previous findings from a phase 3 trial, which showed an increase in annual growth velocity by 1.57 cm/year compared to individual pretreatment growth velocity, our cohort demonstrated a significant increase in growth, as indicated by improvements in height z-scores. Notably, the patients experienced an increase not only in disease-specific z-scores but also in CDC z-scores, which are based on growth data of unaffected children. This suggests that treated patients exhibited catch-up growth in comparison to both untreated children with ACH and unaffected children over the 12-month period. Interestingly, the mean improvement of the height CDC z-score in our cohort was 0.38 ± 0.44 and exaggerated the results of the phase 3 trial of vosoritide, where an increase of 0.24 ± 0.32 (mean ± SD) in the vosoritide group was observed after 52 weeks of treatment. Furthermore, the annual growth velocity in our cohort was elevated compared to disease-specific reference values. Notably, bone age progressed in accordance with the elapsed time.

As expected, the increased height was also associated with an increase in weight z-scores. At the same time, at least after 12 months of therapy, no significant change in body mass index was observed. Therefore, we have no hint that the increase in weight would lead to additional overweight within this cohort.

Previous studies have shown a positive correlation between increased height and a longer 6-minute walking distance [23]. Therefore, it was not surprising that the increased growth observed in our cohort was also accompanied by a notable increase in the 6-minute walking distance. This increase in walking distance could translate into enhanced physical activity. A recent study has shown that higher levels of physical activity in adult patients with ACH are positively associated with several dimensions of health-related quality of life, particularly general health, vitality, and physical functioning [27]. Overall, patients with ACH have shown lower health-related quality of life and higher psychological distress in adulthood compared to the general population [27]. Therefore, it is important to evaluate quality of life already in children with ACH as a standard component in the multidisciplinary management. Recent published data have revealed that persistent growth-promoting effects of vosoritide in children with ACH are accompanied by improvements in physical and social aspects of health-related quality of life [28]. In contrast to that, we did not observe a significant change in quality of life in our cohort; in fact, there was a slight nonsignificant downward trend in the Physical Well-Being dimension according to caregiver responses. However, the responses regarding quality of life varied widely both between and within individuals, making it difficult to draw clear conclusions about the changes after 12 months of vosoritide treatment. Additionally, one has to keep in mind that Savarirayan et al reported an improvement of quality of life after 3 years of treatment and according to changes in z-score [28]. Based on that, we will reassess quality of life continuously during treatment with vosoritide.

In line with the findings from the phase 3 trial, our study did not observe changes in the upper to lower segment body ratio. As suggested by Savarirayan et al [11], such changes might only become evident if treatment is initiated at a younger age than in our study and if the observation period extends beyond 12 months. Long-term studies on the effects and side effects of vosoritide treatment are currently ongoing (NCT03424018, NCT02724228, NCT03989947) and will also assess changes from baseline in body proportion ratios of the extremities as a secondary outcome measurement. Regarding a long-term investigation of vosoritide treatment, it will also be important to examine whether the observed growth acceleration is consistent across multiple years of treatment or diminishes over time, as seen with other pharmacological therapies. For instance, treatment with recombinant GH, which is approved for ACH in Japan, has shown significant catch-up growth during the first 1 to 3 years, which subsequently plateaued after the fourth year [29]. Based on this observation, recombinant GH was never approved for ACH treatment in Europe. Unlike vosoritide, however, GH does not target the pathophysiological mechanisms of ACH.

Importantly, 3 of 32 patients exhibited a decrease in their ACH height z-score. These 3 patients ranged in age from 3.1 to 5.7 years and included 2 males. All were prepubertal and presented with a delayed bone age of more than 1 year. Two patients required cervical decompression in early childhood. One of these patients discontinued treatment with vosoritide after 15 months.

We could not identify any distinguishing characteristics among these 3 children compared to the rest of the cohort that could explain the decrease in height z-scores. It is important to note that, given this was a retrospective analysis, no electronic diary was kept to maintain the daily administration of vosoritide. Routinely, patients and caregivers were asked about missed doses on each visit. Noncompliance with the therapy was not documented in the medical charts of these 3 patients. Considering the burden of the treatment, noncompliance remains a potential contributing factor to the observed treatment failure. Additionally, findings from Hoover-Fong et al suggest a correlation between pretreatment annual growth velocity and the subsequent increase in growth velocity following therapy, with lower baseline growth velocities being associated with greater magnitude of change [30]. In this study, however, there was no pretreatment observation period, preventing the assessment of initial annual growth velocity. Therefore, no conclusions can be drawn regarding these 6 patients that showed a decrease in height z-score.

This study has several limitations. Due to ACH being a rare disease, studies involving patients with ACH are often constrained by small cohort sizes. In this study, the cohort included 34 patients—a relatively large number for a single-center study yet still limited in terms of statistical power. Moreover, the distribution across age groups was uneven. As newer medications are typically administered to older children before younger ones in clinical practice, the number of younger patients in the study was limited. Furthermore, vosoritide treatment is only possible when growth plates are still open, which restricted the inclusion of adolescent patients. Consequently, the numbers of patients in the age groups of ≥2 to <5 years and ≥10 to <16 years were smaller compared to the middle age group.

Another limitation is that, while the data were prospectively recorded in a registry, the analysis was conducted retrospectively. Therefore, missing data like pretreatment growth velocity, measurements of compliance and assessment of pubertal status could not be retrieved. The lack of baseline growth data limits the ability to directly compare our findings with those from the phase 3 trial of vosoritide. In the phase 3 study, a 6-month observation period prior to therapy allowed for intraindividual comparisons of annual growth velocity before and after treatment. In contrast, our study relied on comparisons with reference values rather than individual growth rates prior to therapy [31]. Another limitation is the lack of information regarding the effect of vosoritide on surgical procedures. This is an important factor, especially for the long-term treatment in these children.

Nonetheless, this study has strengths as it presents the first real-world data on the use of vosoritide in children with ACH. Due to the fact that it is a single-center study, all measurements were conducted by the same trained staff with long-time experience in measuring children with growth disorders. Assessments were taken based on a standardized protocol at our center and at predefined timepoints during clinical visits. Therefore, the results can be regarded as first proof that effects presented in the phase 3 trial are reproducable in a routine real-world setting, at least in a high-experienced center for children and adolescents with rare skeletal disorders.

In summary, our findings demonstrate that children with ACH show increased growth after 12 months of treatment with vosoritide. Specifically, our cohort exhibited a higher annual growth velocity compared to disease-specific reference values, along with improvements in individual height z-scores. However, no significant effect was observed on the upper to lower segment body ratio, as assessed by the ratio of sitting height to body height after 12 months. Future research should focus on long-term outcomes and assessment of the potential effects on body proportions and comorbidities. Lastly, identifying potential predictive factors for response seems to be essential to reduce the risk of side effects and improve personalized treatment.

## Data Availability

Some or all datasets generated during and/or analyzed during the current study are not publicly available but are available from the corresponding author on reasonable request.
